# Enhancing treatment decision-making: pilot study of a treatment decision aid in stage IV non-small cell lung cancer

**DOI:** 10.1038/sj.bjc.6604395

**Published:** 2008-05-27

**Authors:** N B Leighl, F A Shepherd, D Zawisza, R L Burkes, R Feld, J Waldron, A Sun, D Payne, A Bezjak, M H N Tattersall

**Affiliations:** 1 Division of Medical Oncology, Princess Margaret Hospital/University Health Network, University of Toronto, Toronto, Ontario, Canada; 2 Department of Radiation Oncology, Princess Margaret Hospital/University Health Network, University of Toronto, Toronto, Ontario, Canada; 3 Department of Cancer Medicine, University of Sydney, Sydney, New South Wales, Australia

**Keywords:** advanced non-small cell lung cancer, treatment decisions, decision aid, systemic therapy, chemotherapy, informed consent

## Abstract

We developed a decision aid (DA) for patients with metastatic non-small cell lung cancer (NSCLC), to better inform patients of their prognosis and treatment options, and facilitate involvement in decision-making. In a pilot study, 20 patients with metastatic NSCLC attending outpatient clinics at a major cancer centre, who had already made a treatment decision, reviewed acceptability of the DA. The median age of the patients was 61 years (range 37–77 years), 35% were male, 20% had a university education, and most (75%) had English as a first language. Most had received chemotherapy, with 65% currently on treatment. Patients were not anxious at baseline and had clear understanding of the goals and toxicity of chemotherapy in advanced NSCLC. After reviewing the DA, patients' anxiety decreased slightly (*P*=0.04) and knowledge scores improved by 25% (*P*<0.001). Most improvements in understanding were of prognosis with and without chemotherapy, although patients still believed advanced NSCLC to be curable. Patients rated the DA highly with respect to information clarity, usefulness and were positive about its use in practice, although 40% found the prognostic information slightly upsetting. The DA for advanced NSCLC is feasible, acceptable to patients and improves understanding of advanced NSCLC without increasing patient anxiety.

Despite advances in treatment for cancer, most patients with metastatic cancer die of their disease, with life expectancy in the range of months. Lung cancer is the most common cause of cancer-related death in North America, with over 200 000 cases diagnosed annually (American Cancer Society, 2006; National Cancer Institute of Canada, 2006). Whereas cancer treatments for metastatic disease may prolong life, improve cancer-related symptoms and cause reduction in tumour size, these effects are not guaranteed. Some patients benefit from treatment, others do not. In the case of systemic therapy for non-small cell lung cancer, the median survival improvement with first-line chemotherapy is approximately 2 months, although up to 70% of patients may have symptom improvement (Lopez *et al*, 1997). Uncertain gains from treatment must be balanced against the likely occurrence of treatment-related side effects, which on occasion, can be life-threatening. Patients who understand their prognosis, treatment options and the potential benefits and risks of those options can make informed treatment decisions in accordance with their personal values.

We developed a decision aid (DA) for patients with metastatic non-small cell lung cancer, to be used during oncology consultations, to facilitate decision-making for advanced lung cancer patients. We then pilot-tested the DA in patients who had already made a treatment decision for a preliminary evaluation of its acceptability for use and explored any potential impact on understanding and anxiety in this experienced group.

## MATERIALS AND METHODS

This study was conducted at a single quaternary cancer centre in Toronto, Canada, with the approval of the institutional research ethics board. All study participants provided written informed consent to participate.

### Decision aid development

#### Information to be presented

Based on the required elements of informed consent (Cassileth *et al*, 1980), studies of advanced lung cancer patient information needs (Gamble, 1998; Davidson *et al*, 1999) and the input of focus groups of advanced lung cancer patients, the following questions, to be addressed through the DA, were identified. (1) What is the diagnosis? (2) What is the patient's prognosis? (3) What are the treatment options? (4) What are the benefits, including survival and quality-of-life effects? (5) What are the potential risks? (6) What is involved for patients pursuing those options, for example schedules, required investigations and hospital visits, routes of administration.

Searches of Medline, the Cochrane Collaboration and American Society of Clinical Oncology websites were performed, (MeSH terms ‘lung cancer’, ‘non-small cell’, ‘randomized’, ‘randomised’, ‘systematic review’, ‘meta-analysis’). An expert panel of oncologists and oncology nurses with lung cancer expertise reviewed and identified standard treatment options accepted by the lung oncology community. Three meta-analyses of trials comparing chemotherapy with supportive care (one updated) (Souquet *et al*, 1993; Marino *et al*, 1994; Non-small Cell Lung Cancer Collaborative Group, 1995; Non-small Cell Lung Cancer Collaborative Group, 2000), and three systematic reviews were identified (Lopez *et al*, 1997; Sorenson *et al*, 2001; Paesmans, 2002). No additional randomised trials of first-line chemotherapy *vs* supportive care were identified. Information on the survival impact of first-line chemotherapy and the effects on quality of life were incorporated into the DA. By patient request, survival and quality of life data from current randomised trials using newer generation chemotherapy were also presented. Data on toxicity of commonly used regimens in the institution were derived from multicentre randomised trials (Kelly *et al*, 2001; Schiller *et al*, 2002). The DA incorporated options of supportive care +/− systemic therapy and clinical trial participation. To help patients integrate their values into the decision-making process, the ‘weigh scale’ values clarification exercise, developed by O'Connor *et al.* (1995), was adapted for use by lung cancer patients and incorporated into the DA.

#### Presentation format

Presentation format was informed by the National Health and Medical Research Council guidelines on presenting evidence to consumers (National Health and Medical Research Council, 1999), current literature (Brundage *et al*, 2005) and focus groups of patients and caregivers. The DA was designed as a 25-page booklet (letter-sized paper) with treatment options, toxicity and survival information illustrated in graphic (as well as numeric and verbal) format for use during the consultation, which patients could then take home, with an audiorecording. An adaptation of Quality-adjusted Time Without Symptoms and Toxicity (Q-TwiST, Gelber *et al*, 1993) was used to illustrate the concept of the trade off between treatment toxicity and potential gains from therapy. Additional information presented included colour-coded calendar schedules of treatment days and days of most likely toxicity (as advised by the expert panel and patient experiences, where possible), a flowchart of different treatment pathways and a list of references. Relevant clinical trials available at the Princess Margaret Hospital with their purpose, design and study sponsors were included, as well as a guide to support and information services. The reading level of the DA was grade 7.8, using the Flesch-Kincaid method (Microsoft Word, Microsoft Corp., Redmond WA, USA).

### Pilot-testing in advanced lung cancer patients

Consecutive patients attending outpatient thoracic oncology clinics at the Princess Margaret Hospital over 4 months were invited to participate in the study after approval by their oncologist and provision of written consent to participate. Inclusion criteria for participation were: (1) diagnosis of metastatic non-small cell lung cancer; (2) age 18 years or older and (3) first-line systemic treatment decision had already been made. Patients were excluded if they were (1) deemed inappropriate by the treating physician for reasons of well-being or anxiety, (2) non-English speaking, (3) undergoing a change in treatment and (4) unable to provide informed consent.

Demographic data were collected by self-report on all patients including age, gender, marital status, occupation, educational level, first language, country of origin, time since diagnosis of metastatic disease and history of prior therapy. Patients were invited to review the in-consultation diagrams and booklet through a structured interview. Feasibility and acceptability, including the amount, length and clarity of information, and usefulness of the DA, were assessed by a 10-item questionnaire adapted from Fiset *et al.* (2000) and Barry *et al.* (1995) to assess acceptability of shared decision-making programmes. Patient anxiety, as another measure of acceptability of the aid, and knowledge were measured using a pre-/post-test design, using the 20-item State-Trait Anxiety Inventory (STAI Form Y) and a 16-item scale adapted from Fiset *et al.* (2000) and Brundage *et al.* (2001). Oncologists were also invited to review the DA and assess feasibility and content validity.

#### Statistical analysis

Frequency data on demographics, feasibility and acceptability were summarised, and pre-/post-anxiety and knowledge scores were compared using a paired samples *t*-test (SPSS Statistical Package). The impact of demographic variables on knowledge scores (gender, educational attainment, English-speaking background, age, prior chemotherapy and baseline anxiety), was explored through *χ*^2^analyses.

## RESULTS

Twenty patients with Stage IV NSCLC were invited to participate, and all consented. Eight patients were screened by research staff and deemed too unwell to participate. No patient was excluded by their physician for anxiety. All were able to review the DA within 25 min (range 15–25 min). The median age of patients was 61 years (range 37–77 years), 35% were male, 20% had a university education and most (75%) had English as a first language (see Table 1). All but one had received chemotherapy, with 65% currently on treatment. After reviewing the aid, 90% felt strongly that they would choose chemotherapy, (if they were to choose again). The patient who had never received treatment was unsure and another who had previously received chemotherapy was leaning towards supportive care alone.

Patients rated the DA highly with respect to clarity of information and usefulness and felt treatment options were presented in a balanced manner. Nineteen (95%) rated everything as clear, one (5%) rated most things as clear; sixteen (80%) rated the DA as very useful and four (20%) as somewhat useful. Most (95%) felt they would have benefited from using the DA in their own treatment decision. All were positive about using the aid, although 40% found the prognostic information slightly upsetting, but anxiety as measured by the STAI did not increase (see Table 2). Patient feedback suggested that although information about treatment benefit is important, the maintenance or promotion of hope despite the prognosis of advanced NSCLC is essential to patients.

Patients were not anxious at baseline (median score 29, range 21–43, maximum possible score 80). After reviewing the DA, patient anxiety decreased slightly (*t*_df19_ −2.199, *P*=0.04). They had clear understanding of the goals and toxicity of chemotherapy in advanced NSCLC prior to reviewing the DA, as expected in a group of experienced patients, and this improved by 25% after reviewing the aid (median 4 of 16 items, t_df19_ 6.472, *P*<0.001). Most improvements in understanding related to prognosis with and without chemotherapy, and supportive care. Despite an explicit statement that current chemotherapy options for metastatic disease were not offered with curative intent, all patients reported that metastatic NSCLC was curable after reviewing the DA. There was no impact of demographic variables on knowledge (gender, educational attainment, English-speaking background, age, prior chemotherapy and baseline anxiety).

## DISCUSSION

This DA, developed to assist patients with advanced NSCLC considering first-line chemotherapy during and after their initial oncology consultation, is feasible, highly acceptable to patients and improves understanding. In this experienced group of patients, the aid helped to add additional information to their current understanding of their illness and did not significantly increase their anxiety, suggesting that the DA may be an acceptable and highly informative tool for newly diagnosed patients, to be evaluated in subsequent trials. All but one patient reported that they would have liked to have reviewed the DA prior to their own treatment decision. Many patients and their families want detailed information about their diagnosis, prognosis, treatment options and to be active participants in the decision-making process (Lobb *et al*, 1999). Involving patients in decision-making may contribute to better health outcomes. However, to be active participants, patients must have accurate information about their illness and treatment options. Whereas internet use has greatly enhanced patient's access to healthcare information, it can be inaccurate or misleading or taken out of the relevant medical context (Wyatt, 1997; Jadad and Gagliardi, 1998). Despite efforts to ensure informed decision-making, it is often incomplete. Oncologists do discuss the goals of systemic therapy with advanced cancer patients, as well as its impact on prognosis and side effects. However, in an analysis of 118 audio-taped oncology consultations with advanced cancer patients, fewer oncologists discuss life expectancy, the patient's treatment preference and alternatives, such as supportive care alone (Gattellari *et al*, 2002). The impact of treatment on patient's quality of life was discussed in only one-third of consultations, and oncologists checked for patient understanding of information in only 10%. Even when all relevant information is discussed with their oncologists, as many as a third of patients misunderstand this information (Gattellari *et al*, 1999). This should not surprise oncologists, given the amount and complexity of information to review, the anxiety and distress associated with a diagnosis of advanced cancer and possible coping strategies, such as denial. As denial is an important contributor to patient's misunderstanding, the clarity of information received from the oncologist is also predictive of understanding. Many patients with advanced disease overestimate their prognosis, and more than half are clearly overoptimistic. For example, Weeks *et al*, (1998) found in a large study of inpatients with advanced cancer that 58% were overly optimistic about their prognosis compared to their physician . These patients were much more likely to request aggressive medical interventions, although their survival was not improved when compared to those who did not pursue aggressive care. In our study, patients failed to report that the goal of treatment was not cure, despite enhanced understanding of potential outcomes and toxicities of systemic therapy after reviewing the DA. It is unclear whether this represents patient denial, misunderstanding of the DA or even misunderstanding of the question asked. Perhaps, rather than a lack of understanding, this is a measure of patient hope, such as the potential for curative therapy to be developed in the future, or even the existence of miracles.

As many as 18% of advanced cancer patients in the United States are receiving chemotherapy within 14 days of death, a number that is increasing over time (Earle *et al*, 2004). Although misunderstanding or denial may contribute to this phenomenon, there are alternate explanations. Cancer patients' valuation of therapeutic benefit differs from that of the general population (Matsuyama *et al*, 2006). Even the well-informed patient may seek aggressive therapy for small gains despite toxicity, which may also contribute to the increase in aggressive end-of-life care. Our study confirms the findings of Matsuyama *et al.* (2006) that receiving realistic information about prognosis is difficult in the setting of advanced cancer. Despite this, it remains important that patients are well informed about their prognosis and realistic outcomes of available treatments, to ensure that their decisions are in keeping with their personal values.

Other DAs for advanced NSCLC patients have been developed. Fiset *et al.* (2000) developed a workbook and an audiotape for metastatic NSCLC patients to take home post-consultation. The aid has been shown to improve patients' knowledge of options and outcomes and reduce decisional conflict. But despite only limited discussion of prognosis with and without chemotherapy, this did upset some patients. Brundage *et al.* (2001) developed an aid using a structured interview to help patients work through trade off exercises to clarify their values for the outcomes of median survival and 1-year or 3-year survival with the addition of chemotherapy to supportive care. Although the majority of patients were able to complete the exercises, this approach may be limited in clinical practice by the amount of time and dedicated personnel required. In the current study, we have developed an aid with additional prognostic information, and connected it to the physician encounter, by including diagrams the health care team can use when reviewing treatment options and chemotherapy teaching in the consultation.

The potential benefits of DAs for cancer patients include enhanced achievement of their information needs, greater understanding of the treatment options, goals, benefits and risks, and greater involvement in their treatment decision-making and cancer care. A meta-analysis of randomised trials of DAs in medicine found that their use produces higher knowledge scores, lower decisional conflict and more active patient participation in decision-making (O'Connor *et al*, 1999). To assess the impact, this DA may have on patient treatment decisions and the decision-making process, a randomised trial is needed. The current pilot study in experienced NSCLC patients, many of whom received chemotherapy, confirms the potential of this tool to enhance current education about advanced NSCLC therapy, despite potential bias in the pilot study sample.

In the setting of advanced cancer, DAs may facilitate the introduction of supportive or palliative care, discussion of life expectancy and setting realistic expectations for treatment outcomes. However, a key challenge for DAs in advanced cancer is to promote or sustain hope, when the goal of treatment is not cure. Despite evolution in the management of advanced NSCLC, progress is slow and treatment outcomes for the majority remain poor. The protection of hope without promoting confusion in advanced NSCLC remains a challenge for patient and oncologist alike.

## Figures and Tables

**Table 1:** Sample characteristics (*n*=20)

*Age*	
Median	61 years
Range	37–77 years
⩾65 years	8 (40%)
	
*Gender*	
Male	7 (35%)
Female	13 (65%)
	
*Marital status*	
Married	14 (70%)
Widowed	4 (20%)
Divorced	1
Single	1
	
*Education*	
High school	6 (30%)
College	10 (50%)
University	4 (20%)
	
*Occupation*	
Professional	4 (20%)
Paraprofessional	4 (20%)
Clerical	10 (50%)
Labourer	2 (10%)
	
English as first language	15 (75%)
Prior medical or allied health training	3 (15%)
	
Prior chemotherapy	19 (95%)
Treatment ongoing	13 (65%)

**Table 2:** Acceptability of the DA (*n*=20)

	***N* (%)**
*Amount of Information*	
About right	17 (85%)
Little more than wanted	3 (15%)
Lot more than wanted	0
Little less than wanted	0
Lot less than wanted	0
	
*Length of DA*	
About right	12 (60%)
Little too long	6 (30%)
Much too long	2 (10%)
	
*Information clarity*	
Everything clear	19 (95%)
Most things clear	1
	
*Helpfulness*	
Very helpful	15 (75%)
Helpful	5 (25%)
	
*Balance between options*	
Balanced	16 (80%)
Slight slant toward chemotherapy	3 (15%)
	
*Appropriateness of DA*	
Very appropriate	16 (80%)
Somewhat appropriate	4 (20%)
	
*Usefulness*	
Very useful	16 (80%)
Somewhat useful	4 (20%)
	
*How do you feel about DA?*	
Very positive	9 (45%)
Generally positive	11 (55%)
	
*Was the information upsetting?*	
Not upsetting	12 (60%)
Slightly upsetting	8 (40%)
	
*Would you like to have seen before you made your decision?*	
Definitely yes	11 (55%)
Yes	8 (40%)
Perhaps	1
