# Supplementary material for: Enhancing treatment decision-making: pilot study of a treatment decision aid in stage IV non-small cell lung cancer
Source: Br J Cancer. 2008 May 27;98(11):1769–73. doi: 10.1038/sj.bjc.6604395 (PMC2410111; doi:10.1038/sj.bjc.6604395)
Supplement: Supplementary Information 1 [file 6604395x1.doc]

**Draft Decision Aid for**

**Patients with**

**Advanced Nonsmall Cell Lung Cancer**

**facing a**

**Treatment Decision**

# Introduction

Your doctor has explained that your cancer, which started in your lung, has spread or has returned in another part of your body. Once cancer has spread, it is called metastatic. There is treatment for your cancer, even though that treatment will not cure your cancer.

Everyone has different needs when it comes to learning about his/her illness, and when it comes to making decisions about treatment. Some people want all the information available about their cancer, while others want to know as little detail as possible. When it comes to making decisions about treatment, some people trust the doctor to decide, while some want to decide for themselves. Others want to share the decision with their doctor. All of these options are normal and acceptable. The best option is the one which best suits **you** and **your** needs.

After talking about your diagnosis with your doctor, there are questions you may wish to address. These include:

### Yes

### No

1. Do you have symptoms from your cancer? You and your doctor may want to watch

how you feel for the next few months. Or you may want to consider treatment now, but there is no definite benefit in starting chemotherapy early if you have no symptoms.

Supportive Care

Anticancer Treatment

Anticancer Treatment

Anticancer Treatment

Anticancer Treatment

Supportive Care

Supportive Care

Supportive Care

Supportive Care

Anticancer Treatment

Anticancer Treatment

**Anticancer Treatment**

1. What treatment approach would you like to take? **+/-** These approaches are explained on

Anticancer Treatment

Anticancer Treatment

Anticancer Treatment

Anticancer Treatment

page 2.

**Standard Treatment**

**Clinical Trial**

1. If you decide to have active treatment, should it be or ?
2. Finally, a second round of chemotherapy may be an option in future, if you and your doctor feel it is appropriate.

Supportive Care

2^nd^ line treatment

**What are the main approaches in initial treatment of my cancer?**

There are 2 main approaches to treatment after you’ve been diagnosed with advanced disease:

1. **Supportive care treatment +/- Radiotherapy**

The aim of this treatment pathway is to keep you as comfortable as possible and to maximize your quality of life and cause as few side effects as possible. Treatment may include pain medications such as morphine, oxygen, and even blood transfusions if they will help you feel better and if you wish to have them. Supportive care does not include chemotherapy treatment, but may include **local anticancer treatment** such as **radiation** to your lung or to a painful area, or even minor surgery to clean out an air passage in your lung (bronchus). It may also include drainage (temporary or permanent) of any fluid around your lung (pleural effusion) to improve your breathing.

2. **Adding Anticancer treatment to Supportive Care +/- Radiotherapy**:

The aim of this treatment pathway is to attack the cancer, often using **chemotherapy**. It may prolong your life, and may help improve your symptoms by shrinking the cancer or stopping its growth, but it may also cause side effects. Not everyone benefits from chemotherapy, and side effects can be severe, even life-threatening. This treatment approach includes **supportive** measures such as pain medicine or oxygen at home, and can include **local treatment** against cancer, such as **radiation** or on an area of the cancer. Please ask your doctor if you would like a tour of the outpatient treatment areas prior to deciding.

**Watch and Wait**

For **people with no symptoms from their cancer**, a **third pathway** is possible—to follow how you feel over the next few months, and consider delaying anticancer treatment until you develop symptoms. Starting treatment now or later doesn’t appear to affect overall survival.^1^ If you and your doctor decide on this option, the rest of the information in this decision aid will not be relevant until you develop symptoms.

**Clinical Trials**

Also, you may hear about clinical trials, or newer experimental treatments available for cancer therapy. If you are interested in learning about clinical trials, be sure to ask your doctor about what trials are available for your type of cancer (see page 20).

**Changing your treatment strategy**

It is important to remember you can always stop one form of treatment and change to another, such as stopping chemotherapy if the side effects are too great and starting supportive care or vice-versa. There is **no** such thing as a **wrong treatment choice**--the choice

which suits you best is the right one, and depends on what is important to you.

**Personal Support**

# Your health care team also wants to assist you and your family in coping with this disease and whichever treatment you choose. Many people living with the diagnosis of advanced colorectal cancer may benefit from support in coping with their illness. This may be from family or friends, or through books, counselling, religious or other spiritual support. Some need support for their family, nursing care at home, and some wish to meet people going through a similar experience. Ask your doctor what support is available–some contacts are included on page 22.

# OVERVIEW OF MANAGEMENT OPTIONS

WAIT AND WATCH

STANDARD CANCER TREATMENT

2^nd^ LINE TREATMENT

ANTICANCER TREATMENT

+

SUPPORTIVE CARE

# Do I have symptoms

*POTENTIAL CHOICES IN THE*

*FUTURE*

# from my cancer?

CLINICAL TRIALS

SUPPORTIVE CARE

SUPPORTIVE CARE

What are your doctor's goals in looking after you?

Doctors have several goals when starting treatment for metastatic cancer.^1^ These include:

1. **Helping you live as long as possible;**
2. **To help improve or relieve any symptoms you may be having because of your cancer;**
3. **Preventing symptoms from cancer for as long as possible;**
4. **Improving your overall well-being (if worse because of your cancer); and**
5. **To promote hope of a positive outcome.**

While your doctor will do his or her best to help you meet some or all of these goals, you should make sure your doctor is aware which goals are most important to you (e.g. your quality of life, or to live as long as possible, or both). This way, you and your doctor can determine which treatment is best for you.

**How does your doctor decide which treatment to recommend?**

**Your doctor takes into account 3 sets of factors when making a treatment recommendation. The first set of factors is about your health.** *Are you fit enough for chemotherapy and side effects? Do you have health*

*problems other than your cancer?*

**The second set of factors relates to your cancer.** *Has it been changing or growing very quickly? Is it causing you*

*symptoms?*

**The third set of factors relates to your values***. What do you want as your therapy? How willing are you to trade off chemotherapy side effects to try and live longer?*

### SUPPORTIVE CARE

# **ALONE**

CHEMOTHERAPY +

SUPPORTIVE CARE

“I feel too sick

to try chemo

“I don’t want chemotherapy”

I want

aggressive treatment

## “I feel well - I can cope with chemo side effects”

“I don’t want any more needles or tests”

“The cancer isn’t causing me any symptoms”

“I want to try and live longer”

**What differences are there between the 2 treatment pathways?**

**Supportive Care Supportive Care +**

**Anticancer Treatment**

Will my **pain** and cancer symptoms Yes **🗸** Yes **🗸**

**be treated**?

Will I be offered interpersonal and spiritual Yes **🗸** Yes **🗸**

support?

Will I receive **chemotherapy**? No **🗴** Yes **🗸**

Will I be offered **radiation** treatment Yes **🗸** Yes **🗸**

or minor **surgery** if it could relieve my

cancer symptoms?

**How often** will I need to come in to About **once every 1 to 3 months** Can vary, but often 2 visits

the hospital **clinic**? and when you have problems **every three weeks**

*plus* possible extra visits for tests

**How often** will I need **blood tests**? This **varies**, but whenever you have symptoms **Varies** from **once a week to every 3** suggesting a blood abnormality, and for some **weeks** (before chemotherapy given) or if doctors, at each visit (**every 1 to 3 months**) you have symptoms suggesting

abnormal blood tests

**How often** will I need **x-rays** or CT **scans**? **Varies** depending on your symptoms, but likely Usually a chest x-ray at each visit, and every 2 to 3 months or less often other scans may be needed every 2 or 3

months (but not everyone needs these).

**What differences are there between the 2 treatment pathways?**

**Supportive Care** **Supportive Care + Anticancer Treatment**

How many drugs are used? Only drugs that you need are used, Usually two chemotherapy drugs are used plus such as pain medication (tablets), and medicine to prevent nausea, in addition to any medicine some people may not need any drugs. you require such as pain medication.

How often do I get treatment? There is no particular schedule. Any The 2-drug combination is given intravenously (IV). medication you need should be taken as Most combinations are given through the intravenous your doctor prescribes. For example, on a single day each week for 2 weeks in a row, pain medication is usually taken daily. followed by a rest week. This 3 week period is called a

“cycle”. Cycles continue for as long as the treatments are helping and if you wish to continue. (The treatment can start on any day of the week).

 Days when you may have nausea or vomiting

 Days when you may be tired, and when your blood counts would be lower than normal

♦ Days to take nausea prevention tablets

The first IV treatment usually takes 4 to 5 hours, as you receive both chemotherapy drugs and fluid (saline) to flush out your system (kidneys). The second IV treatment takes about 30 to 60 minutes, and you receive only one chemotherapy drug.

What will the side effects be? **Supportive Care Supportive Care + Anticancer Treatment**

The only side effects might be from pain Depending on the type of chemotherapy you and medicine (constipation, dry mouth, mild your doctor choose, the side effects may vary.

nausea) or local anticancer treatment (such Some people have few side effects, while others can have as radiation) and would be minor. more severe side effects. Almost all people

notice some effects from chemotherapy. The charts below describe side effects for a commonly used 2-drug combination, vinorelbine and cisplatin.^2^ The percentages refer to how often these effects would be severe, for example, needing to come in to hospital for a few days or longer. If you do get severe side effects, your doctor may **delay your next treatment** by a week, **reduce your dose** of chemotherapy or even **change treatment.**

**What effect will the 2 different treatment pathways have on my cancer?**

The figures below are different ways of showing the information about the effects of treatment (gathered from randomized trials, the highest quality medical evidence)^3-5^

Chemotherapy can help increase the chance of living to 1 year or longer by 10%, compared with supportive care by itself. That means that 10 more people out of 100 will live past 1 year if they all receive chemotherapy. It’s important to remember that everyone is not “average”, and some people have lived 6 years or even longer with this disease. Also it is important to remember that statistics apply to groups of people, and not necessarily an individual like you.

**Supportive Care Supportive Care + Anticancer Treatment**

In **1 year**'s time?

More recent studies suggest that people who are well and receive newer chemotherapy combinations can have a 30% or higher chance of living past 1 year.

Median or “average” Half of people live longer than Half of people live longer

Survival 4 to 6 months, and half of people than 6 to 9 months, and half live that long or less. live that long or less.

**Supportive Care Supportive Care + Anticancer Treatment**

**🚹🚺🚹🚺🚹🚺🚹🚺🚹🚺 15% 🚹🚺🚹🚺🚹🚺🚹🚺🚹🚺 25%**

**🚹🚺🚹🚺🚹🚺🚹🚺🚹🚺 🚹🚺🚹🚺🚹🚺🚹🚺🚹🚺**

**🚹🚺🚹🚺🚹🚺🚹🚺🚹🚺 🚹🚺🚹🚺🚹🚺🚹🚺🚹🚺**

**🚹🚺🚹🚺🚹🚺🚹🚺🚹🚺 🚹🚺🚹🚺🚹🚺🚹🚺🚹🚺**

**🚹🚺🚹🚺🚹🚺🚹🚺🚹🚺 🚹🚺🚹🚺🚹🚺🚹🚺🚹🚺**

**🚹🚺🚹🚺🚹🚺🚹🚺🚹🚺 🚹🚺🚹🚺🚹🚺🚹🚺🚹🚺**

**🚹🚺🚹🚺🚹🚺🚹🚺🚹🚺 🚹🚺🚹🚺🚹🚺🚹🚺🚹🚺**

**🚹🚺🚹🚺🚹🚺🚹🚺🚹🚺 🚹🚺🚹🚺🚹🚺🚹🚺🚹🚺**

**🚹🚺🚹🚺🚹🚺🚹🚺🚹🚺 🚹🚺🚹🚺🚹🚺🚹🚺🚹🚺**

**🚹🚺🚹🚺🚹🚺🚹🚺🚹🚺 🚹🚺🚹🚺🚹🚺🚹🚺🚹🚺**

**🚹 Alive with cancer Newer Chemotherapy Treatments**

**🚹 Died from cancer**

**🚹🚺🚹🚺🚹🚺🚹🚺🚹🚺 30 –35%**

**🚹🚺🚹🚺🚹🚺🚹🚺🚹🚺**

**🚹🚺🚹🚺🚹🚺🚹🚺🚹🚺**

**🚹🚺🚹🚺🚹🚺🚹🚺🚹🚺**

**🚹🚺🚹🚺🚹🚺🚹🚺🚹🚺**

**🚹🚺🚹🚺🚹🚺🚹🚺🚹🚺**

**🚹🚺🚹🚺🚹🚺🚹🚺🚹🚺**

**🚹🚺🚹🚺🚹🚺🚹🚺🚹🚺**

**🚹🚺🚹🚺🚹🚺🚹🚺🚹🚺**

**🚹🚺🚹🚺🚹🚺🚹🚺🚹🚺**

**What impact will the 2 treatment pathways have on my quality of life?**

**1. Supportive Care:** Your doctor will try to relieve your symptoms as best as possible through medication, radiotherapy treatment or minor surgical treatment, as well as involving team members to help you and your family cope with your disease, and help provide social and spiritual support.

**2. Adding Anticancer therapy to Supportive Care:** There are several studies which have shown that adding chemotherapy to supportive care in patients with lung cancer who are well enough to receive treatment, can improve their cancer symptoms, and in some studies, can improve quality of life.^4^ This is despite the side effects of chemotherapy. It is estimated that as many as 70% of people who receive chemotherapy may have an improvement in their symptoms,^3^ such as cough, shortness of breath, pain and improvements in their activity level.

**If your cancer shrinks or does not get worse with chemotherapy and you are having symptoms from your cancer, some aspects of your quality of life may improve with chemotherapy. However some aspects of your quality of life may be worse, depending upon the side effects you have from treatment.**

**Quality of life changes with chemotherapy**

**What are the pros and cons of supportive care, with or without anticancer therapy?**

**Supportive Care** **Adding Chemotherapy Treatment to Supportive Care**

**Pros** + my symptoms will be controlled + may help me live longer

+ I will not have side effects from cancer treatment + may improve my cancer symptoms

+ I will have less interruption in my daily life without + if my symptoms improve, I may be able to use less of other

frequent trips to hospital for treatment medicines such as morphine (which may have side effects)

+ other reasons: ______________________________ + I feel I'm doing something to fight the cancer

+ __________________________________________ + other reasons: ___________________________________

+ _____________________________________________

**Cons** I worry that I’m not doing anything to try and  I will have treatment side effects

control the cancer

 there is no guarantee treatment will work

other reasons: _________________________

 I'll need more tests, needles and IVs if I have chemotherapy

_____________________________________

 inconvenience of frequent visits to hospital

for me and my family

I may have less free time

 I may need additional medicines for treatment side effects

 other reasons: __________________________________

 _____________________________________________

**Arriving at a treatment decision**

Arriving at a decision can be thought of as a series of **7** steps. You have already carried out **3** of these steps:

1. **Understanding your situation**
2. **Learning about your treatment options**
3. **Reviewing the pros and cons of those options**

The next **4** include determining:

1. **how important these pros and cons are to you**
2. **where you are leaning —towards supportive care alone or adding anticancer treatment?**
3. **if you want more information or more discussion with your doctor**
4. **who should make the decision—you, your doctor, or shared between you**

The next page shows examples of how other people view the pros and cons of this decision. This will be followed by your own worksheet^6^, where we will ask you to please list the pros and cons of your options in the boxes, and shade in the boxes according to how important these are to you. Shade in the whole box if the statement is very important, half of the box if it is somewhat important to you, and do not shade at all if the statement is not important to you.

###### We encourage you to share and review this with someone in your family.

# Gertrude's Worksheet

**Weighing up the pros and cons of supportive care versus active treatment**

#### Supportive Care

#### + Anticancer Treatment

#### Supportive Care

**Cons of Adding Anticancer Treatment Pros of Adding anticancer Treatment**

| **Chemotherapy side effects**  Cousin with breast cancer had terrible side effects. | I may live longer I have lived to a good age. |
| --- | --- |
| **Frequent tests, trips to hospital**  I have to take the bus or a taxi to get there. | **My symptoms from cancer may improve**  I don't really have any symptoms right now. |
| **Less free time for myself**  I try to golf every day if I can. | **I feel like I'm fighting the cancer**  I'm not a quitter. |
| **No guarantee treatment will work**  It didn't work for my cousin. | **I may be able to stop my other medications**  I'm not on any other medicines right now. |
| **Other**  I can always start later if I change my mind. | **Other** |

1. **Are there any more questions you would like to ask?**

*Can I start chemotherapy treatment later if I want to?*

6. **Who should make the decision about treatment?**

**🗆 🗆 🗆 🗆 🗆 🗆**

###### I would prefer I prefer to make I prefer that my I prefer that my I prefer to leave I'm not sure

to make the the final decision doctor and I share doctor make the all decisions who should

decision after considering responsibility for final decision, about treatment make the

my doctor's the decision but strongly to my doctor decision

opinion consider my

opinion

1. **Where am I leaning?**

**Supportive Care 🗆 🗆 🗆 🗆 🗆 🗆 🗆 Adding Anticancer Treatment**

# *Tom's Worksheet*

# Weighing up the pros and cons of supportive care versus active treatment

#### Supportive Care

#### Supportive Care

#### + Anticancer Treatment

**Cons of Adding Anticancer Treatment Pros of Adding Anticancer Treatment**

| **Chemotherapy side effects**  They can be bad, but they're only temporary? | I may live longer I have lived a good life. |
| --- | --- |
| **Frequent tests, trips to hospital**  I'm on my own – I can drive myself. | **My symptoms from cancer may improve**  I have some pain in my right side, but it's not bad. |
| **Less free time for myself**  I'm not very active these days anyway. | **I feel like I'm fighting the cancer**  That's very important. |
| **No guarantee treatment will work** | **I may be able to stop my other medications**  I hate taking pills. |
| **Other**  I could have a bad reaction to chemotherapy. | **Other** |

**5. Are there any more questions you would like to ask?**

*Are the side effects of chemotherapy only temporary? How long do they last? Can I stop if I have a bad reaction?*

*Do I have to take other pills with the chemotherapy?*

*Would radiation help my pain instead?*

6. **Who should make the decision about treatment?**

**🗆**  **🗆 🗆 🗆 🗆 🗆**

###### I would prefer I prefer to make I prefer that my I prefer that my I prefer to leave I'm not sure

to make the the final decision doctor and I share doctor make the all decisions who should

decision after considering responsibility for final decision, about treatment make the

my doctor's the decision but strongly to my doctor decision

opinion consider my

opinion

###### **Where am I leaning?**

###### **Supportive Care 🗆 🗆 🗆 🗆 🗆 🗆 🗆 Adding Anticancer Treatment**

# Anna's Worksheet

**Weighing up the pros and cons of supportive care versus active treatment**

#### Supportive Care

#### Supportive Care

#### + Anticancer Treatment

**Cons of Adding Anticancer Treatment Pros of Adding Anticancer Treatment**

| **Chemotherapy side effects** I am strong—I can handle it | I may live longer I have my children to be around for. |
| --- | --- |
| **Frequent tests, trips to hospital**  My husband or I can drive to hospital | **My symptoms from cancer may improve**  I am having a lot of lower back/stomach pain. |
| **Less free time for myself**  I can have free time after treatment is over. | **I feel like I'm fighting the cancer**  I can't give up fighting, because of my kids. |
| **No guarantee treatment will work**  True. | **I may be able to stop my other medications**  The morphine is very constipating. |
| **Other** | **Other**  My brother had chemotherapy and he wasn't too sick with it. |

**5. Are there any more questions you would like to ask?**

*Do I need any more tests?*

*When can I start?*

6. **Who should make the decision about treatment?**

**🗆 🗆 🗆 🗆 🗆 🗆**

###### I would prefer I prefer to make I prefer that my I prefer that my I prefer to leave I'm not sure

to make the the final decision doctor and I share doctor make the all decisions who should

decision after considering responsibility for final decision, about treatment make the

my doctor's the decision but strongly to my doctor decision

###### opinion consider my

opinion

**7. Where am I leaning?**

######

###### **Supportive Care 🗆 🗆 🗆 🗆 🗆 🗆 🗆 Adding Anticancer Treatment**

***Your Worksheet***

**Weighing up the pros and cons of supportive care versus active treatment**

#### Supportive Care

#### Supportive Care

+ Anticancer Treatment

**Cons of Adding Anticancer Treatment Pros of Adding Anticancer Treatment**

| **Chemotherapy side effects** | I may live longer |
| --- | --- |
| **Frequent tests, trips to hospital** | **My symptoms from cancer may improve** |
| **Less free time for myself** | **I feel like I'm fighting the cancer** |
| **No guarantee treatment will work** | **I may be able to stop my other medications** |
| **Other** | **Other** |

1. Are there any more questions you would like to ask?

1. Who should make the decision about treatment?

**🗆 🗆 🗆 🗆 🗆 🗆**

###### I would prefer to I prefer to make I prefer that my I prefer that my I prefer to leave I'm not sure who

make the decision the final decision doctor and I share doctor make the all decisions about should make the

after considering responsibility for final decision, but treatment to my decision.

my doctor's opinion the decision strongly consider doctor.

my opinion.

###### 7. Where am I leaning? **Supportive Care 🗆 🗆 🗆 🗆 🗆 🗆 🗆 Adding Anticancer Treatment**

# REFERENCES

1. Grunfeld EA, Ramirez AJ, Maher EJ, et al. Chemotherapy for advanced breast cancer: what influences oncologists’ decision-making? Br J Cancer 84:1172-2001.
2. Kelly K, Drowley J, Bunn PA, et al: Randomized Phase II Trial of paclitaxel plus carboplatin versus vinorelbine plus cisplatin in the treatment of patients with advanced non-small cell lung cancer: A Southwest Oncology Group Trial. J Clin Oncol 19: 3210-3218, 2001.
3. Lopez PG, Stewart DJ, Newman TE, Evans WK: Chemotherapy in stage IV (metastatic ) non-small-cell lung cancer. Provincial Lung Disease Site Group. Cancer Prev Control 1:18-27, 1997.
4. Sorenson S, Glimelius B, Nygren P, et al: A systematic overview of chemotherapy effects in non-small cell lung cancer. Acta Oncologica 40:327-339, 2001.
5. Non-small Cell Lung Cancer Collaborative Group. Chemotherapy for non-small cell lung cancer (Cochrane Review). In: *The Cochrane* *Library*, Issue 2, 2000. Oxford: Update Software.
6. Adapted from: O'Connor A, Tugwell P, Elmslie T & Wells G. Do decision aids help postmenopausal women considering preventive hormone replacement therapy (HRT)? Med Decis Making 15:433, 1995.

# **Clinical Trials for Patients with Advanced Lung Cancer**

Clinical trials are research studies, and often test how useful new treatments are, or compare them to what is used currently. There are 2 trials currently available to interested patients. If you are interested or wish to learn more about trials, please ask your doctor. Or you can find more information through the National Cancer Institute at <http://www.cancer.gov/clinical_trials>

1. Randomized study of Tarceva versus placebo in combination with gemcitabine and cisplatin

Who can join the trial? People who have **advanced lung cancer** and are well enough to have chemotherapy.

What is it about? Tarceva is a new medication (tablet) which stops cancer growth (for a time) in some people with lung cancer. The cancer experts want to know if adding Tarceva to our usual chemotherapy treatment helps people live longer or feel better.

In this trial, half of people will receive chemotherapy (usual treatment for lung cancer) plus a placebo (tablet with no active medication) and the other half will receive the same chemotherapy plus Tarceva. Who receives placebo and who receives Tarceva is determined by chance (“randomization”).

How do we know which The doctors will follow how people do in the study, looking

way is better? closely at how long people live, their quality of life

and when their cancer starts to grow

Who is providing money The company that makes Tarceva, Roche Pharmaceuticals,

for the study? is providing the money for the chemotherapy, tests you will need and nursing support.

1. Phase 1 study of Iressa plus vinorelbine and cisplatin

Who can join the trial? People who have **advanced lung cancer** and are well enough to have chemotherapy.

What is it about? This study is looking at the safety of adding Iressa, a new medication (tablet) for lung cancer treatment, to chemotherapy. The cancer experts want to know if adding Iressa to our usual chemotherapy treatment is safe, and well tolerated, and how high a dose of Iressa people can take without too many side effects.

In this trial, people will receive chemotherapy (usual treatment for lung cancer) plus a certain dose of Iressa .

How do we know if the The doctors will follow how people do in the study, looking

treatment is safe? closely at side effects, and the effect of the treatment on the cancer.

Who is providing money The company that makes Iressa, Astra Zeneca, is providing

for the study? the money for the chemotherapy, tests you will need and nursing support.

**Support Services for People with Lung Cancer**

Different people have different needs for support. There are a variety of support services available. These are a list of a few support services, and you can find out about more services through them. Also feel free to ask your doctor or nurse for more information.

1. **Patient and Family Library, Princess Margaret Hospital** (main floor)

- resource room and library, open from 9AM to 3PM Monday to Friday
- **CD Rom:** Understanding Lung Cancer

1. **Wellspring, 416-961-1928** (81 Wellesley St East, Toronto ON M4Y 1H6)

- Information Exchange Board with programs and events across Toronto
- **Support Programs**, including coping training for patients and families

1. **Alliance for Lung Cancer Advocacy, Support and Education (ALCASE)**

- provides information support, and developing support groups
- website [www.alcase.org](http://www.alcase.org/) with Canadian site under construction ([www.alcase.ca](http://www.alcase.ca/))

1. **Pamphlets/Booklets**:

- Making Choices: Treatment of Stage IV (metastatic) non-small cell lung cancer. A Decision Aid for patients (Eli Lilly Oncology Company) – a booklet and tape discussing chemotherapy and supportive care

Lung Cancer: What you need to know (Canadian Cancer Society)

- Lung Cancer: An Epidemic? (Janssen-Ortho Company)

- Choices: treatment options for people who have been diagnosed with lung cancer (Bristol-Myers Squib Company)

- Lung Cancer: information for people with lung cancer and their families (Ortho-Biotech Company)

1. **Internet resources**:

Alliance for Lung Cancer Advocacy [www.alcase.org](http://www.alcase.org/)

Support and Education [www.lung.ca](http://www.lung.ca/)

Canadian Cancer Society [www.cancer.ca](http://www.cancer.ca/)

American Cancer Society [www.cancer.org](http://www.cancer.org/)

National Cancer Institute (USA) [www.cancernet.nci.nih.gov](http://www.cancernet.nci.nih.gov/)

1. **Ontario Smoker’s Helpline 1-877-513-5333**

- toll-free number provides free information, advice and support from trained Quit Specialists (advice for those who want to help someone quit smoking also available)

There are also a number of books available about lung cancer. Some popular choices include:

Lung Cancer: Making Sense of Diagnosis, Treatment and Options, Lorraine Johnston; O’Reilly & Associates, 2001.

Living with Lung Cancer: A Guide for Patients and Their Families, Barbara Cox et al; Triad Publishing Company, 1997.

What to Eat if You Have Cancer, Daniella Chace et al; Contemporary Books, 1996.

Your doctor may also have pamphlets available on nutrition, and the clinic nurse or social worker may be able to help with other sources of information and support.
