# Supplementary material for: Enhancing treatment decision-making: pilot study of a treatment decision aid in stage IV non-small cell lung cancer
Source: Br J Cancer. 2008 May 27;98(11):1769–73. doi: 10.1038/sj.bjc.6604395 (PMC2410111; doi:10.1038/sj.bjc.6604395)
Supplement: Supplementary Information 2 [file 6604395x2.doc]

Below are a number of statements about advanced cancer and treatment. Some are true and some are false. Please indicate whether you think the statements are true or not by ticking the appropriate box.

True False Don’t know

1. When cancer is metastatic, this means it has   

spread to another part of the body.

1. Chemotherapy can cure metastatic lung   

cancer.

1. Having chemotherapy is the only option for a   

person with metastatic lung cancer.

d) Most chemotherapy for lung cancer treatment   

makes people lose their hair temporarily.

1. Taking chemotherapy involves regular blood tests   

and visits to the doctor.

1. The average person with metastatic lung   

cancer is not likely to live more than 10 years.

1. A person with metastatic lung cancer can   

live longer than 1 year.

Below are some statements about the impact treatment can have on a person's life span. Please tick the box you think best answers the question.

1. At 6 months, what percentage of people are stable or better if they are on supportive care (no chemotherapy)?

    

0 – 20% 21-40% 41-60% 61-80% 81-100%

1. At 12 months, what percentage of people who take chemotherapy are alive?

    

0 – 20% 21-40% 41-60% 61-80% 81-100%

1. What percentage of people get severe side effects from chemotherapy?

    

0 – 20% 21-40% 41-60% 61-80% 81-100%

**Thank you for filling out this questionnaire.**
